# Efgartigimod for Generalized Myasthenia Gravis and Beyond: A Narrative Review of Its Pharmacological Profile, Clinical Utility, and Expanding Applications

**DOI:** 10.3390/biomedicines13122975

**Published:** 2025-12-04

**Authors:** Ghaith K. Mansour, Leen Alangari, Leen Khosyfan, Reem Alhammad, Ahmad W. Hajjar

**Affiliations:** 1 College of Pharmacy, Alfaisal University, Riyadh 11533, Saudi Arabia; gkmansour@alfaisal.edu; 2 College of Medicine, Princess Nourah Bint Abdulrahman University, Riyadh 11671, Saudi Arabia; 443000398@pnu.edu.sa (L.A.); 441004908@pnu.edu.sa (L.K.); 3 College of Medicine, King Saud University, Riyadh 11472, Saudi Arabia; ralhamad@ksu.edu.sa; 4 College of Medicine, Alfaisal University, Riyadh 11533, Saudi Arabia

**Keywords:** efgartigimod, FcRn antagonist, gMG, autoimmune therapy, IgG modulation

## Abstract

Efgartigimod is a novel neonatal Fc receptor (FcRn) antagonist that reduces pathogenic immunoglobulin G (IgG) autoantibodies, offering a targeted therapeutic approach for generalized myasthenia gravis (gMG) and other antibody-mediated autoimmune diseases. This narrative review synthesizes clinical trial data, pharmacological insights, and real-world evidence to evaluate efgartigimod’s efficacy, safety, and emerging applications. Phase 3 randomized controlled trials and extension studies demonstrate rapid and sustained improvements in muscle strength and patient-reported outcomes with a favorable safety profile, including reduced reliance on corticosteroids and intravenous immunoglobulin (IVIg). Additionally, observational studies highlight its expanding utility in diverse IgG-mediated disorders such as immune thrombocytopenia (ITP) and autoimmune encephalitis. Efgartigimod thus represents a paradigm shift in autoimmune disease management, enabling precision immunomodulation with the potential for broad clinical impact and improved patient quality of life (QOL).

## 1. Introduction and Background on Efgartigimod and Myasthenia Gravis

Myasthenia gravis (MG) represents a quintessential antibody-mediated autoimmune disorder [1]. The incidence of MG ranges from 4.1 to 30 cases per million person-years, with a prevalence rate of 150 to 200 cases per million globally [1]. The pathophysiological hallmark of MG involves the production of pathogenic autoantibodies directed against various proteins essential for neuromuscular signal transmission [2], most commonly the nicotinic acetylcholine receptor (AChR), which comprises approximately 85% of [1]. Less frequently identified autoantibodies include those targeting muscle-specific kinase (MuSK), low-density lipoprotein receptor-related protein 4 (Lrp4), and agrin [1]. While the development of FcRn antagonists has been covered in the recent literature, this narrative review uniquely integrates the latest clinical trial data with emerging real-world evidence, specifically addressing the gap between controlled trial efficacy and clinical practice in diverse populations. We further delineate the comparative positioning of efgartigimod against conventional therapies and emerging competitors, providing a comprehensive update on its expanding utility in neuroinflammatory disorders beyond MG.

## 2. Pathogenesis and the Role of FcRn

**Figure 1:**
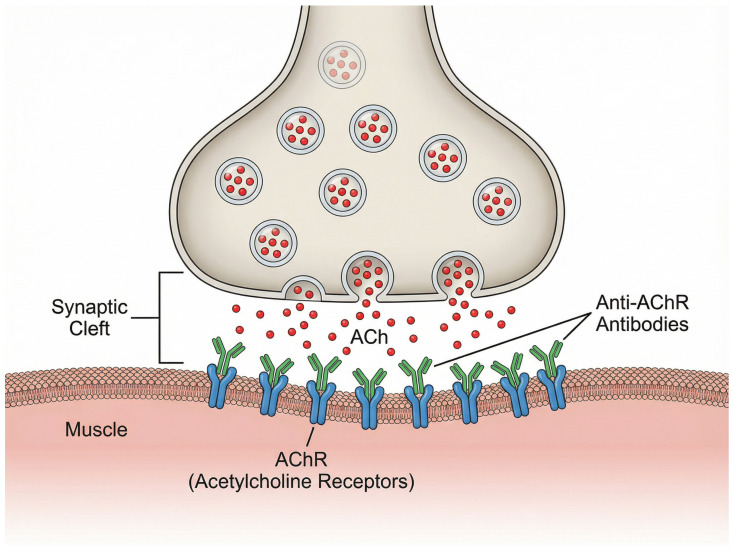
Pathophysiology of Myasthenia Gravis at the Neuromuscular Junction.

FcRn plays a vital role in IgG homeostasis through its recycling mechanism [3]. Under physiological conditions, IgG antibodies undergo pinocytic uptake into cells and bind to FcRn in acidic endosomal compartments. The FcRn then facilitates recycling of IgG back to the cell surface, where they are released into circulation at physiological potential of hydrogen (pH), thereby extending IgG half-life [4]. While this salvage pathway provides advantages in pathogen clearance, it also contributes to the persistence of disease-causing autoantibodies in many autoimmune conditions [4], most notably in gMG. These autoantibodies might disrupt cholinergic transmission between nerve terminals and muscle fibers through multiple mechanisms, including downregulation, destruction, functional blocking, or disruption of AChR clustering in the postsynaptic membrane [1]. The physiological structure of the neuromuscular junction and the interference caused by these pathogenic anti-AChR antibodies are illustrated in Figure 1. Consequently, the persistence of these autoreactive IgG antibodies is essential to the pathophysiology of illness. A promising therapeutic strategy is the inhibition of FcRn by medications (such as efgartigimod). By preventing FcRn from interacting with IgGs, efgartigimod promotes IgG breakdown and quick elimination [5]. The core clinical manifestation of MG is fatigable muscle weakness that characteristically improves with rest and worsens with exertion [6]. The muscle weakness may affect ocular, bulbar, respiratory, and limb muscles, with clinical presentations varying according to the autoantibody type and presence of thymoma [1]. The disease demonstrates a bimodal age distribution with an early peak in the second to third decades, predominantly affecting young women, and a late peak in the sixth to eighth decades, affecting men [6].

Conventional management of MG encompasses symptomatic treatments facilitating neuromuscular transmission, antibody-depleting treatments, and immunotherapeutic strategies [7]. Current standard therapeutic modalities include acetylcholinesterase inhibitors, corticosteroids, nonsteroidal immunosuppressants, IVIg, and plasma exchange [8]. Despite these therapeutic options, many patients with gMG continue to experience insufficient symptom relief and may develop serious adverse effects from existing treatment [9]. This unmet medical need has driven the development of novel targeted therapeutic approaches that provide more rapid onset of action, favorable tolerability profiles, and potential for sustained disease control [10].

While these challenges are evident globally, their impact is especially pronounced in Saudi Arabia and the wider Middle East, where the high prevalence of comorbidities such as diabetes, obesity, and osteoporosis amplifies the risks of adverse events from long-term use of conventional immunosuppressive strategies, including corticosteroids, azathioprine, and related agents [7]. These challenges highlight the need for safer, mechanism-specific approaches. Efgartigimod offers such an option, with clinical trials demonstrating rapid improvements in Myasthenia Gravis Activities of Daily Living (MG-ADL) and Quantitative Myasthenia Gravis (QMG) scores with sustained benefit [11]. Real-world evidence further supports reductions in IVIg dependence, exacerbations, and hospitalizations [12]. For Saudi Arabia, integrating this therapy has the potential to reduce treatment-related toxicity, improve long-term outcomes, and move practice toward more personalized care aligned with contemporary guidance [13].

Efgartigimod alfa (also known as efgartigimod alfa-fcab in the USA) represents the first-in-class FcRn antagonist developed by argenx for the treatment of autoimmune diseases mediated by pathogenic IgG autoantibodies. In December 2021, intravenous (IV) efgartigimod received its first regulatory approval in the United States for the treatment of gMG in adults who are anti-AChR antibody positive [14]. Following the initial FDA approval, efgartigimod has been subsequently approved in Japan in January 2022 for the treatment of gMG patients regardless of antibody status and has entered the preregistration stage in the European Union [14]. The drug has also been approved by Health Canada for patients with AChR antibody-positive gMG [15].

## 3. Chemistry and Formulation of Efgartigomod

Efgartigimod is a human IgG-1 antibody Fc-fragment that serves as a natural ligand of the FcRn [16]. The ABDEG technology involves strategic modifications at five specific amino acid residues within the Fc fragment to dramatically enhance binding affinity to FcRn [17]. These engineered mutations result in increased Fc/FcRn binding at both neutral and acidic pH, creating constitutive blockade of FcRn function and facilitating accelerated clearance of pathogenic antibodies in autoimmune settings [12].

Structurally, it exists as a homodimer composed of two identical polypeptide chains, each containing 227 amino acids, linked via a pair of interchain disulfide bonds. The resulting recombinant protein has an approximate molecular weight of 54 kilodaltons. VYVGART (efgartigimod alfa-fcab) is formulated as a sterile, preservative-free, clear to slightly opalescent, colorless to pale yellow solution, intended for IV infusion following appropriate dilution. It is provided in single-use 20 mL vials, each containing 400 mg of efgartigimod alfa-fcab at a concentration of 20 mg/mL. The excipient profile per milliliter includes 31.6 mg of L-arginine hydrochloride, 5.8 mg of sodium chloride, 2.4 mg of sodium phosphate dibasic anhydrous, 1.1 mg of sodium phosphate monobasic monohydrate, 0.2 mg of polysorbate 80, and sterile water for injection, United States Pharmacopeia (USP), and the formulation is buffered to a pH of 6.7 [18].

The recommended dosage regimen consists of 10 mg/kg administered as an IV infusion once weekly for four consecutive weeks, representing one treatment cycle. Subsequent treatment cycles are initiated based on individualized clinical evaluation and patient response [19]. For patients with a body weight of ≥120 kg, the recommended dosage of VYVGART is 1200 mg per infusion, corresponding to three single-use vials. The initiation of subsequent treatment cycles should be guided by clinical assessment. Notably, the safety and efficacy of administering additional treatment cycles less than 50 days after the initiation of a prior cycle have not been established. In the event of a missed infusion, VYVGART may be administered within a window of up to 3 days following the originally scheduled dose. After administration of the delayed dose, the standard dosing schedule should be resumed to complete the treatment cycle as initially planned [18].

Recent clinical development has also encompassed a subcutaneous (SC) formulation incorporating hyaluronidase (efgartigimod PH20) to enable self-administration and improve patient convenience [20].

Excipients include arginine hydrochloride, sodium chloride, sodium dihydrogen phosphate monohydrate, disodium hydrogen phosphate anhydrous, polysorbate 80 (E433), and water for injections. The product must not be mixed with other medicinal products except for those explicitly specified in the official prescribing information. Incompatibility with unlisted substances has not been evaluated and should be avoided [21].

The unopened vial has a shelf life of 3 years when stored under recommended conditions. Following dilution, chemical and physical in-use stability has been demonstrated for up to 24 h at refrigerated temperatures (2 °C to 8 °C). From a microbiological standpoint, the product should be used immediately after dilution unless aseptic conditions are ensured. If not used immediately, the duration and conditions of in-use storage are the responsibility of the user [21].

VYVGART must be stored in a refrigerator at 2 °C to 8 °C. The product should not be frozen and must remain in its original packaging to protect it from light. After dilution, the same storage precautions apply, and the infusion must be completed within 4 h of removal from refrigeration. The drug is supplied in 20 mL Type I glass vials sealed with a siliconized butyl rubber stopper, an aluminum seal, and a polypropylene flip-off cap. Each carton contains one vial [21].

For preparation, the required volume of VYVGART is withdrawn using aseptic technique and added to an appropriate infusion bag to achieve a total volume of 125 mL using sodium chloride 9 mg/mL (0.9%) solution for injection. Suitable infusion bags include those made of polyethylene (PE), polyvinyl chloride (PVC), ethylene vinyl acetate (EVA), or polyolefin (ethylene/polypropylene copolymer) [21]. Compatible infusion lines include PE, PVC, and polyurethane/polypropylene tubing, and in-line filters composed of polyurethane (PUR) or PVC with polyethersulfone (PES) or polyvinylidene fluoride (PVDF) membranes. The diluted solution should be gently inverted to ensure uniform mixing; vigorous shaking must be avoided. Prior to use, the diluted solution must be visually inspected for particulate matter or discoloration. Any solution exhibiting visible particles, opalescence, or deviation from the expected coloration must not be used. Vials must not be shaken [21].

Dosing should be calculated based on the patient’s actual body weight at 10 mg/kg. The number of vials and volume of diluent required can be calculated as follows: multiply the body weight (up to 120 kg) by 10 mg/kg to determine the total dose; divide the dose by 20 mg/mL to obtain the volume of concentrate; divide that volume by 20 mL to determine the number of vials; subtract the concentrate volume from 125 mL to calculate the required volume of 0.9% sodium chloride for dilution. After preparation, any unused medicinal product or waste material should be disposed of in accordance with local regulatory requirements [21].

VYVGART does not contain any preservatives and must be administered immediately following dilution. If immediate use is not feasible, the diluted solution may be stored under refrigeration at 2 °C to 8 °C (36 °F to 46 °F) for up to 8 h. The solution must be protected from light, must not be frozen, and should be brought to room temperature prior to administration. Importantly, the diluted product should not be warmed by any method other than passive equilibration with ambient air [18].

VYVGART should be administered exclusively via IV infusion by a qualified healthcare professional. Prior to administration, the diluted solution must be visually inspected for particulate matter and discoloration; any solution that appears discolored, opaque, or contains visible foreign particles must be discarded. For administration, the total 125 mL volume of the prepared infusion should be delivered intravenously over one hour through a 0.2-micron in-line filter. Following the infusion, the entire line should be flushed with 0.9% Sodium Chloride Injection, USP, to ensure complete drug delivery [18].

Patients should be monitored during infusion and for at least one hour post-infusion for any clinical signs of hypersensitivity reactions. In the event of a hypersensitivity reaction, infusion must be discontinued immediately and appropriate supportive interventions initiated. Concomitant medications must not be administered through the same infusion line or mixed with VYVGART in the same solution [18].

## 4. Efgartigomod’s Mechanism of Action and Pharmacological Effects

The blockade of FcRn function by efgartigimod disrupts the normal salvage pathway, redirecting IgG antibodies toward lysosomal degradation rather than recycling back to circulation [3]. This mechanism results in reduced serum levels of total IgG, including pathogenic autoantibodies responsible for autoimmune pathology [22].

The mechanism of action of efgartigimod demonstrates selectivity for IgG antibodies while preserving other immunoglobulin classes that do not utilize the FcRn recycling pathway. In clinical studies, efgartigimod administration results in rapid and specific reduction in serum IgG levels, with single administration reducing IgG levels up to 50% and multiple dosing achieving average reductions of 75% from baseline levels [5]. The reduction in total IgG parallels decreases in pathogenic autoantibodies, with AChR antibody levels showing reductions of 57.6% one week after treatment cycle completion [17].

The effects of efgartigimod on IgG levels demonstrate complete reversibility following treatment discontinuation. Approximately 8 weeks after the last administration, IgG levels return to baseline values, indicating that the mechanism does not cause permanent impairment of the FcRn system [5]. This reversible mechanism provides an important safety feature, allowing recovery of normal IgG homeostasis while maintaining the potential for retreatment as clinically indicated. The recovery profile reflects the restoration of normal FcRn function as efgartigimod is cleared from the system, enabling resumption of physiological IgG recycling [23].

## 5. Pharmacokinetics and Pharmacodynamics Behaviors of Efgartigimod

The pharmacokinetic characteristics of efgartigimod reflect its unique mechanism as an FcRn antagonist with target-mediated drug disposition. Following IV administration, efgartigimod demonstrates rapid distribution and exhibits a distinctive biphasic elimination profile characterized by an initial fast decline followed by a relatively slower terminal phase. The initial fast decrease in measurable unbound efgartigimod represents the combined result of free drug clearance and high-affinity target binding to FcRn. The slower terminal pharmacokinetic phase reflects the release of bound drug from the FcRn receptor, indicating that high-affinity target binding protects the drug from elimination and contributes to sustained pharmacodynamic effects [23].

Efgartigimod alfa-fcab demonstrates linear pharmacokinetics across a dose range up to 50 mg/kg (five times the recommended therapeutic dose) with proportional increases in systemic exposure. The estimated volume of distribution ranges from 15 to 20 L, suggesting limited extravascular distribution. Following administration, efgartigimod alfa-fcab is presumed to undergo proteolytic degradation into small peptides and amino acids via endogenous enzymatic pathways. The terminal elimination half-life is approximately 80 to 120 h (3 to 5 days). In healthy subjects receiving a single IV dose of 10 mg/kg, urinary excretion accounted for less than 0.1% of the administered dose, indicating minimal renal clearance [18].

Although formal pharmacokinetic studies have not been conducted in patients with renal impairment, population-level analyses from clinical trials suggest that individuals with mild renal impairment, defined as an estimated glomerular filtration rate of 60–89 mL/min/1.73 m^2^, exhibited approximately 22% higher systemic exposure compared to those with normal renal function (see Use in Specific Populations (8.6) [18]). The implications of this modest increase remain clinically insignificant, but further evaluation in moderate-to-severe impairment is warranted [18].

Dedicated pharmacokinetic studies in patients with hepatic dysfunction are lacking. However, given the expected proteolytic degradation of efgartigimod alfa-fcab, hepatic impairment is not anticipated to substantially alter its pharmacokinetics [18].

No clinical drug–drug interaction studies have been conducted to date. Importantly, efgartigimod alfa-fcab is not metabolized by cytochrome P450 enzymes and is neither an inducer nor an inhibitor of cytochrome P450 enzyme pathways. Consequently, pharmacokinetic interactions with agents that are cytochrome P450 enzyme substrates, inducers, or inhibitors are unlikely. However, due to its high affinity for the FcRn, efgartigimod alfa-fcab may reduce the systemic concentrations of FcRn-binding biologics or therapeutic IgG-based compounds. Caution is advised when co-administering such agents [18].

Population pharmacokinetic modeling has successfully characterized the relationship between efgartigimod exposure and target receptor occupancy across multiple species, demonstrating that drug-induced FcRn receptor occupancy serves as the primary driver of total IgG suppression. The pharmacokinetic behavior shows consistency across different species when adjusted for species-specific FcRn binding affinity and allometric scaling of physiological parameters [23]. In addition, analysis of pooled pharmacokinetic data revealed no clinically meaningful impact of age, sex, or race on drug exposure. However, dedicated pharmacokinetic studies in specific populations remain limited [18].

The pharmacodynamic profile of efgartigimod demonstrates a direct relationship between FcRn receptor occupancy and IgG degradation rate. Increasing target receptor occupancy results in progressive increases in the IgG degradation rate constant, leading to dose-dependent reductions in total IgG levels [23]. Clinical pharmacodynamic studies reveal that efgartigimod produces a rapid onset of action, with measurable IgG reductions beginning within days of the first administration [11]. The magnitude of IgG reduction reaches maximum levels approximately one week following completion of a treatment cycle, with total IgG levels decreasing by an average of 61.3% [17].

Importantly, the reduction in total IgG levels correlates directly with decreases in pathogenic autoantibodies, including anti-AChR antibodies in MG patients [11]. The dose–response relationship for efgartigimod has been characterized through phase 2 and phase 3 clinical studies [11].

Lower doses of 5 mg/kg demonstrated reduced efficacy in achieving target IgG reductions, while the 10 mg/kg dose consistently achieved clinically meaningful reductions in both total IgG and pathogenic autoantibodies. Higher doses did not provide proportional increases in efficacy, supporting the selection of 10 mg/kg as the optimal therapeutic dose [16]. The pharmacokinetic and pharmacodynamic characteristics of efgartigimod demonstrate consistency across diverse patient populations. Clinical studies in Chinese patients with gMG showed comparable efficacy and safety profiles to those observed in global populations, indicating minimal ethnic differences in drug disposition or response [24].

Age-related pharmacokinetic differences appear minimal, with elderly patients demonstrating similar response patterns to younger adults. Patients with multiple comorbidities have shown good tolerability and efficacy, suggesting that common age-related conditions do not significantly impact efgartigimod disposition [25].

## 6. Cost-Effectiveness and Pharmacoeconomics

The economic evaluation of efgartigimod for gMG has been conducted through comprehensive cost-effectiveness analyses employing Markov modeling approaches. [15] These analyses incorporate both direct medical costs and health outcomes measured in quality-adjusted life-years (QALYs) to assess the value proposition of efgartigimod compared to existing treatment options [26]. The economic models account for treatment and administration costs, disease monitoring expenses, complications from chronic corticosteroid use, exacerbation and crisis management costs, adverse event management, and end-of-life care expenses. Health state transition probabilities are derived from clinical trial data, including the ADAPT and ADAPT+ studies, supplemented by network meta-analyses comparing efgartigimod with alternative treatments [15].

A Canadian health technology assessment demonstrated that efgartigimod provides favorable cost-effectiveness compared to chronic IVIg therapy. Over a lifetime horizon, efgartigimod and chronic IVIg were predicted to generate total discounted QALYs of 16.80 and 13.35, respectively, with total discounted costs of $1,913,294 and $2,170,315. This analysis revealed that efgartigimod dominated chronic IVIg with incremental QALYs of 3.45 and cost savings of $257,020 over the lifetime horizon. The dominance profile indicates that efgartigimod provides both superior health outcomes and reduced costs compared to chronic IVIg therapy, representing an economically attractive treatment option [15].

In the United States healthcare context, economic evaluation of efgartigimod compared to conventional therapy revealed different cost-effectiveness profiles. For efgartigimod, lifetime costs and QALYs were calculated at $6,773,000 and 13.22, respectively, compared to conventional therapy costs of $322,000 and 9.98 QALYs. The resulting incremental cost-effectiveness ratio (ICER) was $1,987,000 per QALY gained, which significantly exceeds conventional cost-effectiveness thresholds. When incorporating indirect costs through a modified societal perspective, including productivity losses from patients and caregiver burden, the ICER was reduced to $1,959,000 per QALY gained [26].

Health utility analysis has established significant associations between MG disease severity and health-related QOL measures. The relationship between MG-ADL scores and EQ-5D-5L utility values demonstrates that each unit improvement in MG-ADL leads to a statistically significant utility increase of 0.0233. Individual MG-ADL items contribute differentially to utility values, with the largest impact observed from improvements in brushing teeth/combing hair, rising from a chair, chewing, and breathing. Importantly, patients receiving efgartigimod demonstrated a statistically significant additional utility improvement of 0.0598 compared to placebo-treated patients, indicating benefits beyond those captured by symptom severity scores alone [27].

A network meta-analysis comparing recently approved treatments for gMG revealed that efgartigimod demonstrated the lowest cost per improved outcome (CPIO) among evaluated therapies. Efgartigimod had the lowest number needed to treat (NNT) for achieving clinically meaningful improvements in both QMG and MG-ADL scores. The analysis calculated CPIO values for achieving ≥ 3 points or ≥5 points reductions from baseline in QMG and MG-ADL scores, demonstrating that efgartigimod provided the most favorable economic profile across all assessed efficacy outcomes. This economic advantage stems from the combination of superior efficacy and an acceptable safety profile compared to alternative novel treatments [28].

Analysis of healthcare cost predictors in gMG revealed that specific patient characteristics strongly correlate with high healthcare expenditures. The most important predictors of high costs include MG exacerbations, all-cause inpatient admissions, immunoglobulin use, monoclonal antibody use, and frequency of healthcare encounters. Post-index immunoglobulin use increased the risk of high costs by 261%, while monoclonal antibody use increased costs by 135%. These findings indicate that effective treatments reducing the need for rescue therapies and hospitalizations could provide substantial economic benefits beyond their direct acquisition costs [29].

## 7. Efgartigomod in Toxicological Reports and Safety Profile

The safety profile of efgartigimod has been comprehensively characterized through phase 2 and phase 3 clinical studies encompassing over 400 patient-years of exposure. [19] In the pivotal phase 2 study, efgartigimod was well-tolerated in all patients, with no serious or severe adverse events reported and no relevant changes in vital signs or electrocardiographic findings observed [11].

In the ADAPT+ extension study, 84.8% of participants reported at least one treatment-emergent adverse event, with headache occurring in 24.8% of patients, COVID-19 in 15.2%, and nasopharyngitis in 13.8% [19].

Importantly, the incidence of adverse events remained similar between the efgartigimod and placebo groups in controlled studies, with most events being mild to moderate in severity. Urinary tract infections and upper respiratory tract infections were observed twice as frequently in efgartigimod-treated patients compared to placebo, likely reflecting the mechanism-related reduction in IgG levels [17].

The incidence of serious adverse events has remained low across clinical studies. In the ADAPT+ long-term extension study, similar incidence rates per patient-year of serious adverse events were observed between the original ADAPT study (efgartigimod: 0.11; placebo: 0.29) and ADAPT+ (0.25). Five deaths occurred during the ADAPT+ study, attributed to acute myocardial infarction, COVID-19 pneumonia with septic shock, bacterial pneumonia with myasthenic crisis, malignant lung neoplasm, and an unknown cause with multiple cardiovascular risk factors identified on autopsy. Notably, none of these deaths were considered related to efgartigimod by the investigating physicians [30].

Given the mechanism of action involving IgG reduction, potential increased susceptibility to infections represents a theoretical safety concern [31]. However, clinical studies have not demonstrated a clinically significant increase in serious infections or opportunistic infections [19].

Preclinical studies evaluating the impact of FcRn antagonism on vaccine-induced immune responses demonstrated that efgartigimod treatment did not impair the ability to generate new specific IgG responses or compromise protective immunity against viral challenges. These findings suggest that the reduction in existing IgG levels does not significantly compromise the capacity for new antibody production or immune system function [31].

Safety data in special populations, including elderly patients and those with multiple comorbidities, demonstrate consistent tolerability profiles. A case report of efgartigimod use in a 90-year-old patient with stage IV colon adenocarcinoma showed good tolerability and efficacy, indicating feasibility in complex medical situations [25]. Rare adverse events have been reported in post-marketing surveillance, including cases of Kaposi’s varicelliform eruption and herpetic conjunctivitis [32]. Additionally, isolated reports of antibody overshoot phenomena following treatment discontinuation have been documented, particularly in thymoma-associated MG patients [33].

Post-marketing surveillance through the FDA Adverse Event Reporting System (FAERS) database has provided additional safety insights from real-world clinical use. Analysis of 3040 reports with efgartigimod as the primary suspect drug identified 12,487 adverse events, with the majority being consistent with the known safety profile from clinical trials. The pharmacovigilance analysis confirmed that most adverse events remain mild to moderate in severity, with no unexpected safety signals identified. The real-world safety data support the clinical trial findings and provide reassurance regarding the long-term safety profile of efgartigimod in clinical practice [34].

Limited evidence exists for efgartigimod use during pregnancy and lactation. A case report described safe management of gMG without adverse maternal or fetal outcomes, but more reproductive studies are needed; because FcRn is critical for transplacental IgG transfer, treatment during pregnancy requires individualized risk–benefit assessment and close monitoring [35].

No long-term animal studies have been conducted to evaluate the carcinogenic potential of efgartigimod alfa-fcab. Similarly, genotoxicity has not been assessed in standard mutagenicity assays, and there are currently no data available on the compound’s potential to induce genetic mutations or chromosomal damage. The effects of efgartigimod alfa-fcab on fertility were evaluated in male and female rats administered IV doses of 30 or 100 mg/kg/day prior to and during mating, with continued dosing in females through gestation day 7. These doses represent approximately 3-fold and 10-fold multiples of the recommended human dose (10 mg/kg), based on body weight. No adverse effects on reproductive performance, mating behavior, or fertility parameters were observed in either sex at any dose level [18].

## 8. Patient-Reported Outcomes

The assessment of patient-reported outcomes (PROs) in MG extends beyond traditional physician-rated clinical measures to capture the patient’s subjective experience of disease burden and treatment benefit [36]. The international MyRealWorld-MG digital cohort study of 841 participants across seven countries revealed that the MG Foundation of America (MGFA) class serves as a strong predictor of all aspects of health-related QOL, with patients reporting the most frequent problems in physical domains, including fatigue, problems with daily activities, and mobility restrictions. [36] Importantly, nearly half (47%) of patients reported MG-ADL scores ≥ 3 points, corresponding to an unsatisfactory symptom state that significantly impacts QOL [37].

The pivotal ADAPT phase 3 study, which enrolled 167 patients across multiple regions including Japan and North America, established efgartigimod’s profound impact on MG-ADL, health-related quality of life (HRQoL), and patient-reported QOL through comprehensive assessment using validated instruments including the Myasthenia Gravis Quality of Life 15-item revised scale (MG-QOL15r) and EuroQol 5-Dimensions 5-Levels (EQ-5D-5L) [38]. In AChR antibody-positive participants, efgartigimod produced statistically significant improvements in both MG-QOL15r and EQ-5D-5L utility scores as early as the first week of treatment in both initial and repeat treatment cycles [38]. The improvements demonstrated substantial durability, being maintained for up to four weeks during the follow-up portion of treatment cycles. [38]

The pattern of QOL improvements paralleled changes in IgG levels, with correlational analyses demonstrating consistency between health-related QOL measures and clinical efficacy outcomes [38]. This correlation validates the clinical meaningfulness of the observed improvements and suggests that efgartigimod’s mechanism of reducing pathogenic autoantibodies translates directly into patient-perceived benefit beyond mere symptom reduction [38].

Indirect treatment comparison between efgartigimod and ravulizumab using matching-adjusted methodology revealed superior QOL outcomes for efgartigimod across multiple timepoints [39]. For MG-QOL15r, efgartigimod demonstrated statistically significant improvement compared with ravulizumab over 26 weeks with a mean difference of −52.6 points (95% confidence interval: −103.0 to −2.3), at week 4 with a mean difference of −4.0 points (−6.6 to −1.4), and at time of best response with a mean difference of −3.9 points (−6.7 to −1.1) [39], providing faster and more pronounced improvements in patient-reported QOL compared to alternative novel therapies [39].

Real-world studies have consistently confirmed the QOL benefits observed in clinical trials. A multicenter Chinese cohort study of 61 patients demonstrated that clinically meaningful improvement was rapidly achieved in 97% of patients at a mean of 1.3 ± 0.7 weeks following efgartigimod initiation. By week 12, patients experienced substantial reductions in MG-ADL scores with sustained improvements maintained throughout the follow-up period [40].

Japanese real-world experience from a single-center series of 16 patients confirmed these findings, with 71% of patients achieving QMG responder status (≥3 points reduction) after one treatment cycle. The rapid onset of patient-reported improvement was particularly notable in patients with severe disease, including those with MGFA class V disease and those refractory to complement therapy [41].

Case reports of efgartigimod use in myasthenic crisis have documented remarkable improvements in patient-reported outcomes even in the most severely affected patients. A 57-year-old patient with therapy-refractory myasthenic crisis demonstrated significant improvement within 48 h of efgartigimod administration, as evidenced by reduced MG-ADL and QMG scores that translated into meaningful functional recovery. These cases suggest that efgartigimod’s QOL benefits extend to the most critically ill patients who have failed standard therapies [42]. 

## 9. Efgartigimod in the Broader Autoimmune Landscape

The success of efgartigimod in MG has catalyzed extensive investigation across the broader spectrum of IgG-mediated autoimmune disorders. Primary ITP represents the most advanced application beyond MG, with the phase 3 ADVANCE study and its open-label extension ADVANCE+ providing compelling evidence for sustained therapeutic benefit. In this predominantly refractory patient population with a mean time since ITP diagnosis of 10.6 years, efgartigimod demonstrated clinically meaningful platelet responses with durable efficacy maintained through multiple treatment cycles [43].

The mechanism of action in ITP mirrors that observed in MG, with efgartigimod inducing rapid reductions in total IgG levels, associated with clinically relevant increases in platelet counts. Importantly, 46% of efgartigimod-treated patients versus 25% of placebo recipients achieved platelet counts ≥50 × 10^9^/L on at least two occasions, while 38% versus 0% maintained these levels for at least 10 cumulative days. The accompanying reduction in bleeding episodes provides clinical validation for the therapeutic approach across different autoimmune contexts [16]. Long-term follow-up data from ADVANCE+ confirms the sustainability of therapeutic benefit with repeated efgartigimod cycles, establishing FcRn antagonism as a viable long-term treatment strategy for ITP patients who have failed multiple conventional therapies. The consistent safety profile observed across extended treatment periods supports the broader applicability of this therapeutic approach to chronic autoimmune conditions requiring maintenance therapy [43].

Emerging case series have documented remarkable therapeutic responses with efgartigimod in autoimmune encephalitis, expanding its potential applications to neuroinflammatory disorders beyond the neuromuscular junction. Three patients with distinct forms of autoimmune encephalitis—anti-GABABR, anti-LGI1, and anti-NMDAR encephalitis—demonstrated rapid and sustained clinical improvement following efgartigimod treatment. The speed of response, with marked symptomatic relief observed within days of treatment initiation, suggests that FcRn blockade may provide superior kinetics compared to traditional immunosuppressive approaches [44]. These cases are particularly significant given the limitations of current first-line and second-line treatments for autoimmune encephalitis, which often provide incomplete responses and carry substantial toxicity burdens [44]. The ability of efgartigimod to rapidly reduce pathogenic antibodies directed against neuronal cell-surface antigens opens new therapeutic possibilities for patients with treatment-refractory autoimmune encephalitis [44]. Individual case reports have confirmed efficacy in anti-LGI1-associated autoimmune encephalitis, further supporting the broad applicability of FcRn antagonism across different antibody-mediated neurological syndromes [45].

The successful application of efgartigimod to neuromyelitis optica spectrum disorder (NMOSD) represents another significant expansion of its therapeutic utility. A case report of add-on efgartigimod therapy in acute anti-aquaporin-4 (AQP4) antibody-positive NMOSD demonstrated rapid clinical improvement and successful treatment of an acute attack. This application is particularly noteworthy given the severity of NMOSD attacks and the limited therapeutic options available for acute management [46]. The rapid onset of efgartigimod’s effects makes it particularly suitable in acute settings like myasthenic crises and acute NMOSD treatment, where prompt reduction in pathogenic antibodies may limit tissue damage and improve functional outcomes [46,47]. The case report provides proof-of-concept evidence that FcRn antagonism may serve as an effective rescue therapy for NMOSD patients who fail to respond adequately to conventional acute treatments [46].

Efgartigimod’s efficacy extends to inflammatory myopathies, with case reports demonstrating successful treatment of immune-mediated necrotizing myopathy (IMNM). Seven patients with refractory IMNM showed significant clinical improvement following efgartigimod treatment, with the therapy appearing to shorten disease duration and minimize chronic myopathic features. This application suggests that promoting degradation of endogenous IgG may be broadly effective across different inflammatory muscle diseases [48].

The therapeutic benefit observed in acute motor axonal neuropathy (AMAN), a severe variant of Guillain–Barré syndrome, further demonstrates efgartigimod’s potential in treating peripheral nerve disorders. A case report documented significant improvement in both clinical symptoms and electromyographic findings following efgartigimod treatment in a patient with suboptimal recovery after conventional therapy with IVIg and plasma exchange. This success in AMAN suggests that FcRn blockade may benefit patients with various forms of antibody-mediated peripheral neuropathies [49].

The potential application of efgartigimod to autoimmune bullous diseases represents an important frontier in dermatological therapeutics. Preclinical studies in bullous pemphigoid models have demonstrated that FcRn inhibition with murine Fc fragment IgG2c-ABDEG significantly reduced pathogenic antibody levels and ameliorated skin inflammation compared to control treatments. These findings suggest that efgartigimod may provide therapeutic benefit for patients with pemphigus and bullous pemphigoid who have failed conventional immunosuppressive therapies [50]. The demonstration that FcRn blockade can ameliorate antibody-mediated skin blistering provides important proof-of-concept evidence for clinical trials in autoimmune bullous diseases [50]. Current clinical development programs are investigating efgartigimod in pemphigus and bullous pemphigoid, potentially expanding treatment options for these often refractory dermatological conditions [14].

The potential application of efgartigimod to post-COVID-19 syndrome represents an innovative therapeutic approach based on emerging understanding of autoimmune mechanisms in long COVID. The hypothesis that post-COVID-19 syndrome may result from the development of autoreactive IgG antibodies causing inflammation and tissue injury provides a rationale for FcRn blockade therapy. Efgartigimod’s demonstrated safety profile and mechanism of reducing pathogenic autoantibodies make it an attractive candidate for investigation in this emerging indication [51].

The clinical development pipeline for efgartigimod includes chronic inflammatory demyelinating polyneuropathy (CIDP), a condition where pathogenic antibodies contribute to demyelination and axonal damage. The rationale for FcRn blockade in CIDP mirrors its established mechanism in other autoimmune neurological disorders, with the potential to reduce pathogenic antibodies while preserving physiological immune function [14]. Additional autoimmune conditions under investigation include autoimmune myositis, systemic lupus erythematosus, and various organ-specific autoimmune disorders where pathogenic IgG antibodies play central pathophysiological roles [14]. The disease-agnostic mechanism of FcRn blockade provides a platform approach that could potentially benefit multiple autoimmune conditions, representing a paradigm shift from disease-specific to mechanism-specific therapeutics [14], which could simplify treatment algorithms and provide consistent therapeutic options across different organ systems [52]. The role of FcRn in antigen-presenting cells adds additional complexity to its therapeutic targeting, with potential implications for immune tolerance and autoimmune disease perpetuation [52]. Understanding these broader immunological effects will be crucial for optimizing efgartigimod’s therapeutic applications and identifying patient populations most likely to benefit from FcRn blockade [52].

The accumulated clinical experience with efgartigimod across multiple autoimmune diseases positions it as the most clinically validated FcRn antagonist, with established efficacy and safety data that inform its broader therapeutic applications. As additional clinical trials across various autoimmune conditions progress, efgartigimod’s role as a versatile platform therapy is likely to expand, potentially revolutionizing the treatment landscape for antibody-mediated autoimmune diseases [53].

## 10. Integrating Efgartigimod into International and Regional Guidelines

Management of MG requires individualized strategies that reflect both evidence-based recommendations and the therapeutic options available in each region. Pyridostigmine remains the cornerstone of symptomatic therapy for gMG, while corticosteroids are generally introduced when immunosuppression becomes necessary or when symptoms are inadequately controlled by acetylcholinesterase inhibition [54]. If corticosteroids are not tolerated or fail to achieve sufficient disease control, steroid-sparing immunosuppressants such as azathioprine, mycophenolate mofetil, or cyclosporine are recommended, with treatment selection guided by patient comorbidities and individual response [55]. Current MGFA guidelines (2021) also advise thymectomy for patients younger than 50 years with generalized, AChR antibody-positive MG, while in older patients the decision is influenced by disease severity and surgical risk [56]. IVIG and plasma exchange remain essential for acute indications such as myasthenic crisis or for rapid stabilization in preoperative or life-threatening scenarios [55].

The therapeutic landscape has shifted significantly with the introduction of biologic agents. The European Academy of Neurology (EAN) now recommends FcRn antagonists, including efgartigimod, as add-on options for patients with moderate-to-severe gMG who remain inadequately controlled with corticosteroids and/or conventional immunosuppressants [13]. Similarly, the 2023 Neurological Research and Practice consensus endorses efgartigimod for anti-AChR-positive gMG when rapid steroid-sparing effects are needed or when conventional therapy fails [57]. These guidelines emphasize a broader paradigm shift toward mechanism-specific therapies, including efgartigimod and ravulizumab, designed to minimize long-term immunosuppressive burden while offering faster symptomatic improvement [57].

Current practice in the Middle East region still prioritizes symptomatic therapy with pyridostigmine, followed by corticosteroids and non-steroidal immunosuppressants such as azathioprine or mycophenolate; biologics are generally introduced after inadequate response to these approaches [58]. Importantly, efgartigimod is indicated for adults with gMG who are anti-AChR antibody positive and have not responded adequately to standard therapy [20]. This population represents one of the most difficult groups to manage in daily practice, as they remain symptomatic despite conventional strategies and are at high risk of treatment-related toxicity [19]. Beyond trial evidence, real-world data support its role as a maintenance therapy, particularly for patients tapering off corticosteroids or conventional immunosuppressants and for those with comorbidities limiting steroid use [59]. While its higher acquisition cost presents a barrier, recent pharmacoeconomic models suggest that reduced hospitalizations, avoidance of crisis interventions, and prevention of steroid-related complications may offset long-term costs, supporting consideration of earlier integration into treatment algorithms [60].

## 11. Clinical Development of Efgartigimod in gMG

The clinical development of efgartigimod in gMG has followed a structured progression from early-phase studies to pivotal phase 3 trials. Initial phase 1 studies in healthy volunteers established safety, tolerability, and pharmacokinetic properties, showing robust dose-dependent reductions in circulating IgG without significant adverse effects, thereby providing a rationale for subsequent evaluation in autoimmune populations [5].

The ADAPT trial (NCT03669588) was multicenter, randomized, double-blind, and placebo-controlled, enrolling adults with AChR antibody-positive gMG across. Patients received either efgartigimod or placebo over four 28-day treatment cycles. The trial achieved its primary endpoint, with 68% of efgartigimod-treated patients classified as MG-ADL responders compared with 30% in the placebo arm. Secondary endpoints also favored efgartigimod, with significant improvements in QMG and MGC scores [61].

Long-term efficacy and safety were further characterized in the ADAPT+ extension study (NCT03770403), an open-label trial that allowed continued treatment tailored to clinical need. Interim results confirmed sustained clinical benefit and consistent IgG reduction over repeated cycles [19].

In parallel, other studies, including ADAPT-SC+ and ADAPT-SERON, expanded investigation into underrepresented MG subgroups, such as seronegative and anti-MuSK-positive patients. Preliminary data suggest improvements in MG-ADL and other outcomes, supporting broader therapeutic use [11]. Additional ongoing studies include NCT04650854, which investigates the role of efgartigimod in steroid-sparing treatment regimens, and NCT04735432, which compares the pharmacokinetics and pharmacodynamics of SC versus IV administration in patients with gMG [62,63].

## 12. Clinical Efficacy Versus Safety and Tolerability of Efgartigimod in the Management of gMG

Efgartigimod, a first-in-class FcRn antagonist, has consistently demonstrated strong efficacy alongside an acceptable safety profile across clinical trials and real-world studies in gMG. The most pronounced benefits appeared within 3–5 weeks of treatment initiation and were sustained for up to eight weeks. These outcomes correlated with reductions in total IgG, confirming the pharmacodynamic rationale of FcRn blockade [38].

Real-world findings reinforce these trial data. In Japan’s JAMG-R registry, 62% of 34 patients achieved ≥2 points MG-ADL improvement after one treatment cycle, including those with MuSK-positive and seronegative disease groups often excluded from randomized trials [59]. Notably, MuSK-positive patients had an 83% response rate and showed the largest reductions in MG-ADL scores, suggesting a particularly strong effect in this subgroup. Mean MG-ADL scores declined from 10.5 to 6.9 (*p* = 0.003), with parallel improvements in MG-QOL15r (*p* = 0.007) and MG Composite (*p* = 0.004) scores. Importantly, steroid-sparing effects were observed: prednisolone doses were reduced in seven patients over 26 weeks, with some achieving minimal manifestations on ≤5 mg/day [60]. Longitudinal extension data from ADAPT-SC+ confirmed durable efficacy with repeat cycles [20,24].

In the JAMG-R registry, 13 adverse events were reported in 11 patients, mostly headache, diarrhea, and mild fatigue; none required discontinuation [59]. Importantly, COVID-19 infections occurred in some patients during therapy but did not trigger MG exacerbations requiring acute intervention, suggesting that FcRn blockade does not substantially compromise host defense. Comparative studies in elderly gMG populations have also highlighted a favorable safety profile versus IVIG, with fewer systemic reactions and no thromboembolic complications [64].

Emerging evidence also supports a role in myasthenic crisis. Case series report rapid stabilization and symptom resolution in patients refractory to IVIG or plasma exchange who were subsequently treated with efgartigimod [65]. Additional observational data suggest that patients previously reliant on fast-acting rescue therapies reduced or eliminated their need for such interventions once transitioned to efgartigimod [59].

Of note, efficacy does not appear to depend on the absolute degree of IgG reduction. Analyses from real-world registries found no correlation between the magnitude of IgG decline and clinical response, highlighting the importance of tailoring dosing intervals to symptomatic benefit. [59] Similarly, response rates did not differ significantly by age, sex, disease duration, or baseline severity, reinforcing the broad therapeutic applicability of the drug [59]. However, efgartigimod demonstrates distinct advantages over these conventional therapies. Unlike corticosteroids, which carry high risks of immunosuppression and many adverse events [47], efgartigimod induces rapid improvement, often within 1 week of the treatment administration [38]. While the ADAPT trial did not primarily investigate steroid withdrawal, real-world evidence has highlighted efgartigimod’s utility to reduce steroid requirements. In the Japanese JAMG-R registry, reductions in prednisolone dosage were achievable in patients undergoing maintenance efgartigimod therapy, with some achieving minimal manifestation status on ≤5 mg/day [59]. Furthermore, efficacy extends to populations underrepresented in the initial trials. Although the ADAPT trial focused heavily on AChR-positive patients, real-world data suggest substantial benefit in MuSK-antibody-positive patients and those with seronegative MG [59].

## 13. Short- and Long-Term Clinical Outcomes of Efgartigimod Use in gMG

Rapid reduction in circulating IgG has emerged as a central short-term mechanism of action for efgartigimod in gMG. In a randomized, double-blind, placebo-controlled phase 2 study, efgartigimod induced a mean IgG reduction of 63.7% by day 11, sustained for at least three weeks. These pharmacodynamic changes closely paralleled early improvements in MG-ADL and QMG scores, with clinical benefit evident within the first treatment cycle [11]. Real-world evidence complements these findings. A recent case report of very-late-onset gMG described marked clinical improvement within one week of efgartigimod initiation, including resolution of bulbar weakness and ocular symptoms, highlighting the drug’s rapid onset of action even in elderly patients with comorbidities [66]. The ADAPT-SC trial demonstrated that SC efgartigimod PH20 achieves a comparable magnitude and speed of IgG reduction and symptomatic improvement as IV delivery while maintaining comparable efficacy and safety profiles, thereby expanding administration flexibility without sacrificing [20]. Pharmacodynamic studies in healthy Chinese volunteers further confirmed rapid, dose-dependent IgG reductions with both IV and SC formulations, alongside favorable safety and consistent pharmacokinetics across routes [67]. Long-term extension results from ADAPT+ reinforce the repeatability of these early effects [19]. In the ADAPT+ open-label extension, 145 participants (111 AChR-antibody positive, 34 AChR-antibody negative) received efgartigimod for a mean of 548 days, with up to 17 treatment cycles and a total exposure of 217.6 participant-years. Among AChR-positive patients, more than 90% achieved a clinically meaningful improvement in MG-ADL scores at some point in each of the first 10 cycles. Similarly, 69.4–91.3% achieved improvements in QMG scores across seven measured cycles. Benefits often appeared within one week of starting a cycle and coincided with reductions in total IgG and AChR antibody levels. In those with at least one year of follow-up, the mean annualized treatment rate was 4.7 cycles per year (median 5.0; range 0.5–7.6), supporting an individualized, as-needed retreatment approach. No new safety signals emerged. Results of ADAPT+ reinforced the substantial clinical improvements seen in ADAPT and support its long-term safety, tolerability, and efficacy, as well as an individualized dosing regimen for treatment of gMG [19]. This data addresses a critical clinical question regarding the frequency of administration: there is no fixed “number of rounds.” Instead, treatment is cyclical and individualized, with retreatment triggered by clinical symptom recrudescence. Most patients require approximately 4 to 5 cycles per year to maintain stability [19].

A primary safety consideration regarding this mechanism is the consequence of long-term IgG suppression. Clinical data indicate that while total IgG levels drop significantly (approx. 60–70%) [13], other immunoglobulins (i.e., IgA, IgM, IgE, and IgD) are preserved [19]. Theoretically, this could increase infection risk; however, the ADAPT+ study did not reveal an increase in serious infections [19]. Importantly, the specific humoral response to vaccines remains largely intact, as the mechanism depletes circulating IgG but does not ablate B-cell plasma precursors or prevent the generation of new antibodies [19]. In addition, a large real-world study found that after starting efgartigimod, 84–97% of patients were able to discontinue IVIG, while hospitalizations, MG exacerbations, and myasthenic crises decreased by 63%, 68%, and 71%, respectively. This reduction in crisis events and hospital utilization highlights the ability of efgartigimod to maintain stability over time while reducing reliance on high-burden therapies such as IVIG or plasma exchange [12].

## 14. Comparative Efficacy and Clinical Profile of Other FcRn Antagonists

It is important to contextualize efgartigimod within the broader landscape of FcRn-targeting therapies. While efgartigimod is an Fc fragment, other agents such as rozanolixizumab, nipocalimab, and batoclimab are full-length monoclonal antibodies targeting FcRn [13]. In a recent network meta-analysis, this class of drugs generally demonstrated superior effective response rates compared to complement and B-cell inhibitors [68]. Specifically, Rozanolixizumab exhibited a rapid onset of action, showing significant improvement over placebo as early as one week after initiation, achieving a standardized mean difference (SMD) of −1.80; 95% CI [−3.29, −0.31], mirroring the fast-acting profile of Efgartigimod [68]. Batoclimab also displayed strong efficacy, achieving SMD of −4.58 [−6.05, −3.10] for maximized therapeutic response, ranking it among the most effective agents [68]. Zilucoplan also showed a high efficacy, achieving SMD of −2.08 [−3.57, −0.59] for maximized therapeutic response, ranking it among the most effective agents [68]. Clinical selection among these agents may be influenced by administration routes, as Rozanolixizumab and Batoclimab were administered subcutaneously in the analyzed trials, offering an alternative to the IV administration used for Efgartigimod and Nipocalimab [68]. A shared limitation observed across all these targeted drugs is that none maintained significant efficacy four weeks after treatment discontinuation. For instance, at four weeks post-dosing, the efficacy of both efgartigimod (SMD −1.70) and rozanolixizumab (SMD −1.20) was not statistically different from placebo [68]. Consequently, while efgartigimod is well-established, agents like rozanolixizumab and batoclimab offer comparable maximized efficacy with distinct SC delivery options that may benefit specific patient populations [68]. the key clinical trials and real-world studies establishing the efficacy and safety profile of efgartigimod are summarized in Table 1.

## 15. Conclusions

Efgartigimod is a significant development in the treatment of gMG, confirming the powerful therapeutic target of FcRn, and pathogenic IgG autoantibodies are significantly reduced as a direct result of that. Pivotal trials and empirical data regularly show long-lasting gains in muscle strength tests (MG-ADL, QMG) and patient QOL. Efgartigimod, which has a good safety record, helps change the way that gMG and many IgG-mediated diseases are treated, offering strong pharmacoeconomic potential by reducing hospitalizations and the need for corticosteroids and IVIg. Its broad potential is highlighted by strong findings in ITP and new evidence in autoimmune encephalitis and NMOSD.

## Figures and Tables

**Table 1:** Summary of Key Clinical Trials and Real-World Studies Evaluating Efgartigimod in gMG.

Study	Design	Population	Key Outcomes
Phase 2	Randomized Double-blinded Placebo-controlled	AChR+ gMG	63.7% IgG reduction; improvement in QMG and MG-ADL.
ADAPT (Phase 3)	Multi-center Randomized Double-blinded Placebo-controlled	AChR+ gMG	68% MG-ADL responders vs. 30% placebo; improvement in QMG and MG-ADL.
ADAPT+	Open-label Extension	AChR+/− gMG	Sustained efficacy over multiple cycles; consistent safety profile.
ADAPT-SC	Non-inferiority	gMG (SC vs. IV)	Subcutaneous formulation demonstrated non-inferior pharmacodynamics to IV.
Real-World (Japan)	Observational (JAMG-R registry)	Diverse gMG groups (MuSK+ and seronegative patients)	average efficacy of 62% MuSK+ has the highest efficacy of 83+.

## Data Availability

The original contributions presented in the study are included in the article; further inquiries can be directed to the corresponding authors.

## Abbreviations

The following abbreviations are used in this manuscript:

AChR	acetylcholine receptors
AMAN	acute motor axonal neuropathy
AQP4	Aquaporin-4
CIDP	chronic inflammatory demyelinating polyneuropathy
CPIO	cost per improved outcome
EAN	European Academy of Neurology
EQ-5D-5L	EuroQol 5-Dimensions 5-Levels
EVA	ethylene vinyl acetate
FAERS	FDA Adverse Event Reporting System
FcRn	The neonatal Fc receptor
gMG	generalized myasthenia gravis
HRQoL	health-related quality of life
ICER	incremental cost-effectiveness ratio
IgG	immunoglobulin G
IMNM	immune-mediated necrotizing myopathy
ITP	immune thrombocytopenia
IV	intravenous
IVIg	intravenous immunoglobulin
Lrp4	lipoprotein receptor-related protein 4
MG	Myasthenia gravis
MG-ADL	Myasthenia Gravis Activities of Daily Living
MG-QOL15r	Myasthenia Gravis Quality of Life 15-item revised scale
MGFA	MG Foundation of America
MuSK	muscle-specific kinase
NMOSD	neuromyelitis optica spectrum disorder
NNT	number needed to treat
PE	polyethylene
PES	polyethersulfone
pH	potential of hydrogen
PROs	patient-reported outcomes
PUR	polyurethane
PVC	polyvinyl chloride
PVDF	polyvinylidene fluoride
QMG	Quantitative Myasthenia Gravis
QOL	quality of life
SC	subcutaneous
SMD	standardized mean difference
USP	United States Pharmacopeia
